# Texture-based residual stress analysis of laser powder bed fused Inconel 718 parts

**DOI:** 10.1107/S1600576723004855

**Published:** 2023-06-30

**Authors:** Jakob Schröder, Alexander Evans, Vladimir Luzin, Guilherme Abreu Faria, Sebastian Degener, Efthymios Polatidis, Jan Čapek, Arne Kromm, Gleb Dovzhenko, Giovanni Bruno

**Affiliations:** a Bundesanstalt für Materialforschung und -prüfung, Unter den Eichen 87, 12205 Berlin, Germany; b Australian Nuclear Science and Technology Organisation, New Illawara Road, Lucas Heights, NSW 2234, Australia; c Helmholtz-Zentrum Hereon, Max-Planck-Strasse 1, 21502 Geesthacht, Germany; d Laboratory for Neutron Scattering and Imaging, Paul Scherrer Institut, Forschungsstrasse 111, Villigen 5232, Switzerland; e Universität Potsdam, Institut für Physik und Astronomie, Karl-Liebknecht-Strasse 24-25, 14476 Potsdam, Germany; Montanuniversität Leoben, Austria

**Keywords:** additive manufacturing, electron backscattered diffraction, principal stress, residual stress

## Abstract

Although layer-based additive manufacturing methods such as laser powder bed fusion (PBF-LB) offer an immense geometrical freedom in design, they are typically subject to a build-up of internal stress (*i.e.* thermal stress) during manufacturing. As a consequence, significant residual stress (RS) is retained in the final part as a footprint of these internal stresses. Furthermore, localized melting and solidification inherently induce columnar-type grain growth accompanied by crystallographic texture. Although diffraction-based methods are commonly used to determine the RS distribution in PBF-LB parts, such features pose metrological challenges in their application. In theory, preferred grain orientation invalidates the hypothesis of isotropic material behavior underlying the common methods to determine RS. In this work, more refined methods are employed to determine RS in PBF-LB/M/IN718 prisms, based on crystallographic texture data. In fact, the employment of direction-dependent elastic constants (*i.e.* stress factors) for the calculation of RS results in insignificant differences from conventional approaches based on the hypothesis of isotropic mechanical properties. It can be concluded that this result is directly linked to the fact that the {311} lattice planes typically used for RS analysis in nickel-based alloys have high multiplicity and less strong texture intensities compared with other lattice planes. It is also found that the length of the laser scan vectors determines the surface RS distribution in prisms prior to their removal from the baseplate. On removal from the baseplate the surface RS considerably relaxes and/or redistributes; a combination of the geometry and the scanning strategy dictates the sub-surface RS distribution.

## Abstract 2

In this article, a texture-based characterization of surface, sub-surface and bulk residual stress in laser powder bed fused Inconel 718 alloy is carried out. It is shown that, in the case of this nickel-based superalloy, the texture affects the residual stress determination only when it has sufficient strength.

## 1. Introduction

Layer-wise additive manufacturing methods such as laser powder bed fusion (PBF-LB) have attracted major interest from both academia and industry within the past decade; this interest is based on the immense geometrical design flexibility in the manufacturing of dense parts in a single manufacturing step (Attaran, 2017 ▸). In fact, the aerospace and gas turbine industry sectors demand complex geometries to increase the efficiency of lightweight construction in high-temperature applications. Further, the geometrical freedom enables the design of sophisticated internal cooling geometries in such parts. Owing to its excellent weldability (Lingenfelter, 1989 ▸) paired with its potential in high-temperature applications up to 650 °C (Collier *et al.*, 1988 ▸), the alloy Inconel 718 (denoted IN718) is an established candidate for PBF-LB processing (Volpato *et al.*, 2022 ▸). IN718 is a niobium-, aluminium- and titanium-containing precipitation-hardenable Ni–Cr–Fe–Mo-based superalloy. Its high strength is achieved by the precipitation of γ′′ (Ni_3_Nb, tetragonal *D*0_22_ crystal structure) and γ′ [Ni_3_(Al,Ti), cubic *L*1_2_ crystal structure] phases during aging heat treatments (Cozar & Pineau, 1973 ▸).

However, the layer-wise nature of the PBF-LB process has certain drawbacks that undermine the applicability of the technique: Manufactured parts may suffer from defect formation such as porosity, caused by either gas inclusions or lack of fusion (Foster *et al.*, 2018 ▸). Another inherent problem of the technique is the significant surface roughness (Foster *et al.*, 2018 ▸) of the parts. Although the formation of defects can nowadays be greatly reduced by the selection of appropriate process parameters (Foster *et al.*, 2018 ▸), the surface finish remains a critical aspect for engineering applications (Kasperovich *et al.*, 2021 ▸). The localized melting and solidification mechanisms of the layer-wise technique also inevitably induce large internal stresses during manufacturing (Kruth *et al.*, 2004 ▸; Mercelis & Kruth, 2006 ▸). These are based on the temperature gradient mechanism in combination with the thermal contraction (*i.e.* shrinkage) during cooling of the previous layer, due to mechanical constraint by the substrate plate (Mercelis & Kruth, 2006 ▸; Kruth *et al.*, 2004 ▸; Ulbricht *et al.*, 2020 ▸). In extreme cases, the internal stresses may lead to cracking or delamination during production (Yadroitsev & Yadroitsava, 2015 ▸). In most cases, residual stress (RS) of high magnitude is retained in as-built parts as a footprint of these internal stresses during manufacturing (Schröder, Evans *et al.*, 2021 ▸).

Diffraction-based methods allow the non-destructive determination of the RS distribution of full parts. In principle, lattice spacings (*d^hkl^*) are measured and subsequently used to calculate a lattice strain by comparing them with a stress-free reference value (*d*_0_^*hkl*^). In the case of laboratory X-ray diffraction (XRD) experiments, plane stress can be assumed, *i.e.* the normal stress component vanishes within the penetration depth of the radiation, and a precise knowledge of *d*_0_^*hkl*^ is not required (Spieß *et al.*, 2009 ▸). However, whenever triaxiality of the stress state cannot be excluded, a precise knowledge of *d*_0_^*hkl*^ is indispensable (Withers *et al.*, 2007 ▸), in particular when using penetrant radiation such as neutrons (well suited to the determination of 3D stress fields).

With knowledge of the relationship between elastic lattice strains and macroscopic stress provided by the diffraction-elastic constants (DECs) (Gnäupel-Herold *et al.*, 2012 ▸), RS can be determined from measured strains on the basis of Hooke’s law (Hauk, 1997 ▸). For anisotropic crystals the DECs depend on the *hkl* reflection used to measure the lattice spacing (Gnäupel-Herold *et al.*, 2012 ▸). The DECs can be either determined experimentally or, more commonly, calculated from single-crystal elastic tensor data of the material of interest (Hauk, 1997 ▸). In the past, several grain-interaction models for polycrystalline aggregates have been developed to calculate such DECs from single-crystal data. To name a few, these include the models of isostrain (Voigt, 1889 ▸) and iso­stress (Reuss, 1929 ▸), the average suggested by Hill (1952 ▸), or the Kröner model (Kröner, 1958 ▸) based on the solution of the Eshelby inclusion problem (Eshelby, 1957 ▸). Apart from the Kröner model, preferred grain orientation and grain-to-grain interactions are neglected in these models (Gnäupel-Herold *et al.*, 2012 ▸). However, from Eshelby’s theory (Eshelby, 1961 ▸) it is known that the strain/stress response of a single grain depends on the elastic properties and shape of the surrounding grains (Gnäupel-Herold *et al.*, 2012 ▸). The formulation of Hooke’s law in the form by Dölle & Hauk (1978 ▸, 1979 ▸) overcomes the problem and considers the preferred orientation by introducing the stress factors.

If one wants to select an appropriate model for the calculation of the DECs, it is commonly accepted that the Kröner model provides a reasonable agreement to experimental data for equiaxed polycrystalline IN718 (Schröder, Mishurova *et al.*, 2021 ▸) and IN625 (Wang *et al.*, 2016 ▸). However, another consequence of the localized melting and solidification during the PBF-LB process is the columnar grain growth as reviewed by Volpato *et al.* (2022 ▸). In such cases, it has been experimentally shown that the Reuss model represents the materials behavior for PBF-LB/M/IN718 more accurately (Schröder *et al.*, 2022 ▸; Schröder, Mishurova *et al.*, 2021 ▸). In fact, the usage of DECs based on the Kröner model may lead to RS exceeding the yield strength of as-built PBF-LB/M/IN718 (Pant *et al.*, 2020 ▸; Serrano-Munoz, Fritsch *et al.*, 2021 ▸). Additionally, strong crystallographic textures are characteristic for PBF-LB/M/IN718 (Gokcekaya *et al.*, 2021 ▸), since the f.c.c. crystals grow along the 〈100〉 directions (Chalmers, 1964 ▸). On the one hand, this dependence of the texture on the heat flow allows the texture to be tailored by choosing appropriate scanning strategies and beam parameters (Gokcekaya *et al.*, 2021). On the other hand, the presence of texture requires the usage of the stress factors for the determination of RS. Yet, in the open literature it is common to neglect crystallographic texture when determining RS in PBF-LB/M/IN718. Beyond that, the validity of the general assumption that the directions of principal strain/stress are governed by the main geometrical axes should be additionally questioned (Mishurova, Serrano-Munoz *et al.*, 2020 ▸).

It becomes clear that several metrological challenges of the RS determination in PBF-LB/M/IN718 need to be tackled. In this article the strain and RS distribution in as-built PBF-LB/M/IN718 prisms (manufactured with two different scan strategies) will be determined using a combined approach of laboratory X-ray, high-energy synchrotron and neutron diffraction. These investigations are carried out on material identical to that used in the *in situ* loading studies reported by Schröder *et al.* (2022 ▸). Hence for the isotropic case, the DECs are known to be well predicted by Reuss for the 311 reflection, which mitigates one of the key unknowns for the accurate RS determination. The distribution of sub-surface principal strain and stress is evaluated by strain pole figures and a subsequent eigenvalue decomposition considering texture-based stress factors. Finally, the RS calculations encompassing the crystallographic texture of the two scan strategies are compared with approaches neglecting the presence of texture. Some metrological consequences for RS determination in PBF-LB/M/IN718 prisms are discussed.

## 2. Material and methods

### 2.1. Sample manufacturing

**Figure 1:**
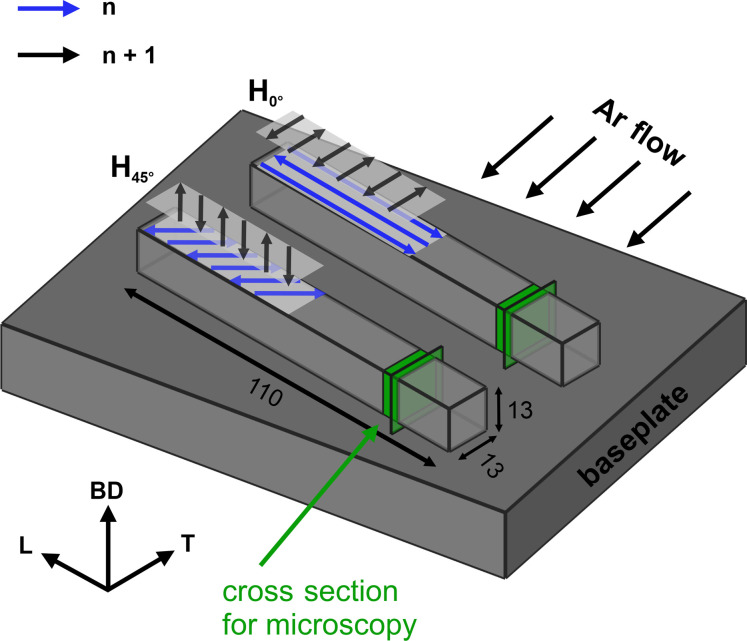
Schematic of the specimens H_0°_ and H_45°_ with their scanning pattern (for layers *n* and *n* + 1) and the extracted cross sections used for microstructural analysis.

The subjects of this study are horizontally built PBF-LB/M/IN718 prisms (110 × 13 × 13 mm^3^) manufactured using an SLM 280 (SLM Solutions Group AG, Lübeck, Germany). The specimens were manufactured with their longest direction within the build plane but tilted by 12° with respect to the build plate edges (Fig. 1 ▸). The baseplate was pre-heated to 200°C and the processing parameters suggested by SLM Solutions were applied: laser power *P* = 350 W, scanning velocity *v* = 800 mm s^−1^, spot size diameter of 0.08 mm defocused by 4 mm and hatch spacing *h* = 0.15 mm. Two different scanning strategies with an interlayer rotation of 90° were applied to produce the specimens (Fig. 1 ▸): in the first variant, the scanning tracks were aligned parallel to the specimen edges (H_0°_), whereas the scanning pattern was rotated by 45° relative to the prism edges for the second variant (H_45°_). The specimens were all used in the as-built state (*i.e.* no heat treatments were applied).

### 2.2. Microstructural analysis

#### 2.2.1. Electron backscattered diffraction

As depicted in Fig. 1 ▸, BD–T (build–transverse directions) cross sections were extracted from sister specimens for microstructural analysis. These cross sections were ground to 1200 grit with SiC abrasive paper followed by subsequent 9, 3 and 1 µm polishing steps. The final polishing step was performed using a 0.04 µm active oxide polishing suspension (OPS, Struers GmbH, Crinitz, Germany). The samples were then mounted in an LEO 1530VP (Carl Zeiss Microscopy GmbH, Oberkochen, Germany) scanning electron microscope, equipped with an electron backscatter Bruker Nano e^−^-Flash HD 5030 detector (Bruker Corporation, Billerica, USA). For the electron backscattered diffraction (EBSD) analysis, the sample was tilted by 70° and kept at a working distance of approximately 18 mm. The acceleration voltage of the electron beam was 20 kV. In essence, for the bulk microstructure an area of 4 × 3 mm^2^ was probed over an 800 × 600 pixel map, *i.e.* with a pixel size of 5 µm. In contrast, the near surface maps were acquired at a higher magnification (250×) with a pixel size of 1.5 µm, *i.e.* over an approximate probed area of 1.2 × 0.9 mm^2^. For data acquisition and indexing the *ESPRIT* (version 1.94) package from Bruker Nano was used. For data post-processing, the open-source *MTEX* toolbox (Bachmann *et al.*, 2011 ▸) installed within MATLAB (The MathWorks Inc., Natick, USA) was utilized. A misorientation angle of 10° was used as the threshold to define high-angle grain boundaries, whereby grains containing fewer than ten pixels were excluded from the analysis.

The grain boundaries were then smoothed using the default kernel (25 iterations). In addition, non-indexed pixels were filled by their nearest neighbor and denoising was performed using a variational spline filter.

#### 2.2.2. Neutron diffraction texture measurements

The bulk texture measurements were performed at the KOWARI strain scanner located at the Australian Nuclear Science and Technology Organisation (ANSTO) in Lucas Heights. For the measurements, cylinders with a diameter and a height of 8 mm were extracted from the center of the threaded region of the H_0°_ and H_45°_ tensile specimens (see Schröder *et al.*, 2022 ▸). The neutron wavelength of 1.4 Å was selected from the 400 reflection of the Si monochromator. With the cylinders fully immersed in the beam, measurements were run with an approximate 3 × 3 (°) mesh (in φ–χ space) over the intervals χ [0, 90]° and φ [0, 360]°. Three detector positions 2θ = 43°, 2θ = 67° and 2θ = 82° corresponding to the 111, 200, 220 and 311 reflections were selected with an acquisition time of ∼2 s. Data post-processing and analysis were performed using *MTEX*. First, the pole figures were normalized; subsequently, orientation distribution functions (ODFs) were calculated using a half-width of 5°. These ODFs were exported into *ISODEC* (Gnäupel-Herold, 2012 ▸). From these ODFs, the {200}, {220}, {111} and {311} pole figures were calculated. Furthermore, the strength of these textures can be quantified by the texture index *J*_ODF_ [equation (1)] as implemented in *MTEX* (Mainprice *et al.*, 2015 ▸). The orientation distribution function can be described as the function *f*(*g*). In this context, the texture index *J*_ODF_ can be defined as the integral of *f*(*g*)^2^ over the rotationally invariant volume element d*g*: 

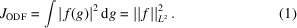

This definition involves the square of *f*, which type of functional is called an *L* ^2^-norm (Mainprice *et al.*, 2015 ▸). For a uniform distribution, *J*_ODF_ returns a value of 1. For a single orientation, it becomes an infinitely large value (Mainprice *et al.*, 2015 ▸).

### 2.3. Texture-based RS analysis

RS analysis by diffraction-based methods rests on Bragg’s law (Bragg & Bragg, 1913 ▸). The lattice spacing *d^hkl^* can be effectively used as a strain gauge. From the comparison between the measured *d^hkl^* and a reference lattice spacing *d*_0_^*hkl*^, the strain can be calculated as the relative difference (Withers *et al.*, 2007 ▸). In this regard, Hooke’s law can be written in the special form of Dölle & Hauk (1978 ▸, 1979 ▸) to determine the macroscopic RS 〈σ_*ij*_〉 from lattice spacings *d^hkl^* [equation (2)]:

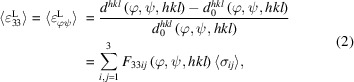

where subscript 33 denotes the laboratory direction 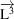 . *F*_33*ij*_(φ, ψ, *hkl*) are the stress factors introduced by Dölle & Hauk (1978 ▸, 1979 ▸), who assigned them the term *F_ij_*, thereby formally missing their fourth rank tensor character. Deriving from the definition, Mishurova, Bruno *et al.* (2020 ▸) showed that the notation is somewhat imprecise, as in fact in the literature ɛ often replaces ɛ_33_, and *F_ij_* is effectively taken as a second rank tensor. For a material without a preferred orientation, the stress factors are independent of the measurement directions ψ, φ; thus they become linear combinations of the DECs *s*_1_ and 1/2*s*_2_ (Hauk, 1997 ▸). However, in the presence of a preferred crystallographic orientation, the *F*_33*ij*_(φ, ψ, *hkl*) depend on the measurement directions. Similar to the DECs, the stress factors can be either directly measured by *in situ* tests or calculated from single-crystal elastic properties using grain-interaction models – note that Gnäupel-Herold *et al.* (2012 ▸) also used the misleading notation *F_ij_*, while properly defining the stress factors. In the latter case, the ODF is required to account for the crystallographic texture of the material studied (Behnken & Hauk, 1991 ▸).

**Figure 2:**
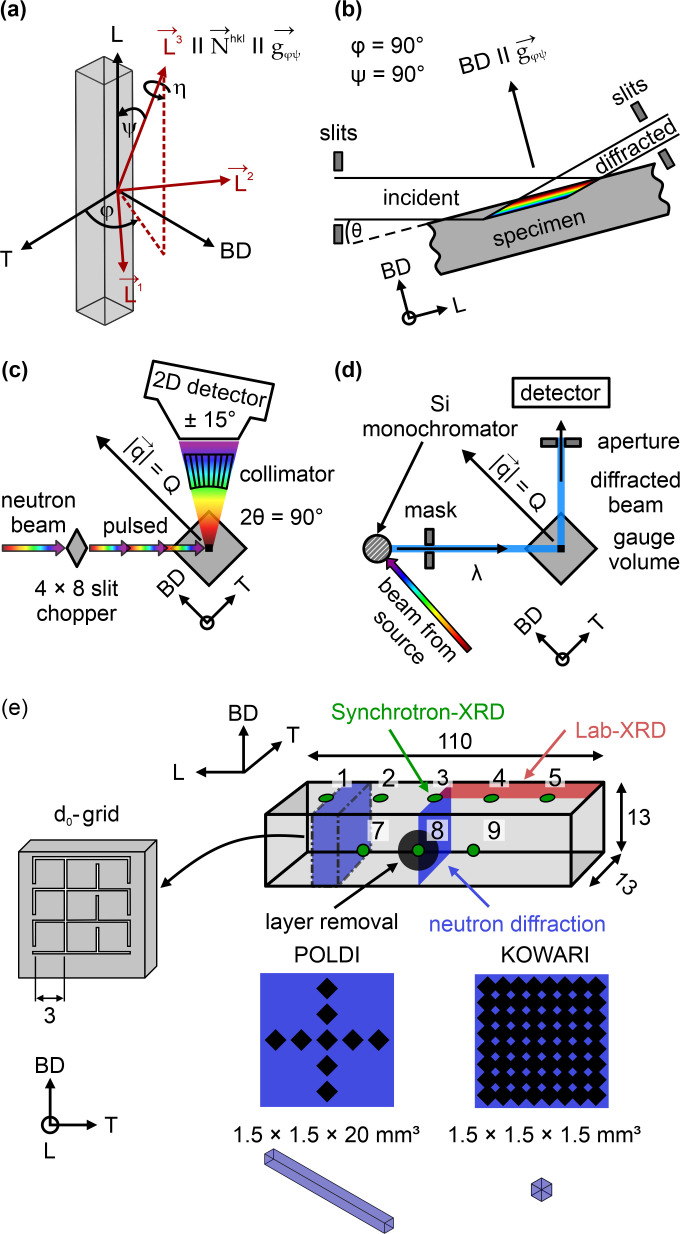
Schematic of the measurement principles: (*a*) reference coordinate system, (*b*) energy-dispersive synchrotron XRD, (*c*) pulse overlap TOF neutron diffraction at POLDI, (*d*) monochromatic neutron diffraction at KOWARI and (*e*) measurement positions for the characterization of RS with an extracted *d*_0_-grid from a sister specimen.

In this study, the texture-dependent *F*_33*ij*_(φ, ψ, *hkl*) were calculated with the software *ISODEC* (version 3.0; Gnäupel-Herold, 2012 ▸) on the basis of the Reuss model (Reuss, 1929 ▸) using the single-crystal elastic constants of IN718 (*c*_11_ = 242.35 GPa, *c*_12_ = 139.73 GPa, *c*_44_ = 104.44 GPa) reported by Haldipur *et al.* (2004 ▸). For the orientation relationships used in this study, see Fig. 2 ▸(*a*).

#### 2.3.1. Laboratory X-ray diffraction

The surface RS measurements were performed with an Xstress G3 diffractometer (StressTech, Vaajakoski, Finland) at Bundesanstalt für Materialforschung und -prüfung (BAM, Berlin, Germany). The system operates in modified χ-mode (see standard DIN EN 15305: 2009–01: *Non-destructive testing – Test method for RS analysis by XRD*) using two position-sensitive detectors, which are calibrated using copper powder. For all measurements, a Ø2 mm collimator and an acquisition time of 5 s were used. For detailed information on the measurement conditions, see Table S1 of the supporting information. The measurement plane for the surface measurements of the top surface (for H_0°_ and H_45°_) is shown in Fig. 2 ▸(*e*). These measurements were performed pre- and post-removal of the specimens from the baseplate to determine the redistribution of the surface RS. Data analysis was performed in the software *Xtronic* using a Pearson VII function to fit the diffraction peaks and determine the *d* ^311^ values. As the classic analysis of the sin^2^ψ method data does not allow the incorporation of texture, the calculation of the RS was performed by the matrix method (also referred to as the generalized sin^2^ψ method) reported by Ortner (2009 ▸, 2011 ▸, 2014 ▸). For all measurements, the overdetermined system of linear equations [see equation (2)] was solved using the generalized linear model as implemented in *statsmodels.api* within Python (Seabold & Perktold, 2010 ▸). Since the penetration depth of the Mn *K*α radiation is at the maximum of ∼5 µm, the out-of-plane stresses were disregarded for the top (σ_*i*-BD_ = 0) and side (σ_*i*-T_ = 0) surfaces. Furthermore, the measurements carry the assumptions that the measured directions (φ = 0°, 90°) are principal, so that the in-plane shear components vanish. As for surface measurements a precise knowledge of *d*_0_^*hkl*^ is not required, the tensor equation (1) can be written in *d^hkl^* form. This treatment yields an overdetermined set of linear equations with the unknowns *d*_0_^311^, σ_TD_ and σ_LD_ (top); and *d*_0_^311^, σ_LD_ and σ_BD_ (side).

#### 2.3.2. Electrolytic layer removal

Incremental electrolytic layer removal was performed (after removal from the baseplate) at the side surfaces of H_0°_ and H_45°_, as indicated in Fig. 2 ▸(*e*). A Kristall 650 electrolytic polishing device (ATM Qness GmbH, Mammelzen, Germany) was used, operated at a voltage of 30 V and a current of 2 A with a circular polishing diameter of 9 mm. The solution used for electropolishing consisted of 550 ml of saturated saline solution, 150 ml of water, 200 ml of ethyl­ene glycol and 100 ml of ethanol. The depth after each removal increment was measured using an ID-C series 543-471B dial indicator (Mitutoyo Corporation, Kawasaki, Japan) with an accuracy of ±3 µm.

#### 2.3.3. Synchrotron X-ray diffraction

The synchrotron XRD measurements were performed at the white beam engineering materials science beamline P61A at the Deutsches Elektronen-Synchrotron (DESY) in Hamburg, Germany [for details see Farla *et al.* (2022 ▸)]. A greatly simplified illustration of the basic instrument principle is shown in Fig. 2 ▸(*b*). Prior to the measurements, a diffraction angle of 2θ ≃ 11.946° was calibrated using NIST silicon powder. The specimens, mounted in an Eulerian cradle, were scanned in χ-mode using the energy-dispersive detector in the horizontal diffraction plane (ψ = χ − 90° for the detector in the horizontal diffraction plane). The specimens were measured at the top and side surfaces according to Fig. 2 ▸(*e*). The acquisition time varied between 10 and 20 s up to ψ = 60° and was increased to 20–40 s between ψ = 60 and 80°. The incoming beam was narrowed by the vertical and horizontal slits to a 0.5 × 0.5 mm^2^ cross section. In the diffracted beam, the slits narrowed the beam to 26 × 26 µm^2^ (for further details on the measurement conditions, see Table S1). Peak fitting was performed in the open-source software *P61A:Viewer* developed at the P61A beamline, using a pseudo-Voigt function. Peaks under 100 counts were excluded from the analysis. The diffraction angle used was approximately 12°, giving a penetration depth of 311 of τ_0_ ≃ 30 µm. As a consequence, stress triaxiality should not be neglected, though its influence on the obtained stress values is expected to be low.

The overdetermined set of linear equations was solved using the mean value of all measured *d* ^311^ as stress-free reference *d*_0_^311^. Afterwards an eigenvalue decomposition was performed to determine the principal directions represented by the eigenvectors **v**_1_, **v**_2_ and **v**_3_, and eigenvalues σ′_L_, σ′_T_ and σ′_BD_. All calculations were repeated 10000 times, selecting a random value within the 95% confidence interval of the least-squares solution to estimate an error band for the principal stress directions and magnitudes. The procedure is described in more detail by Fritsch *et al.* (2021 ▸). Although the choice of *d*_0_^311^ as the mean value of all measured *d* ^311^ values remains somewhat arbitrary, it may affect the absolute values of the principal stresses but not the principal stress directions.

#### 2.3.4. Time-of-flight neutron diffraction

Bulk *d*_0_^*hkl*^ and strain measurements were performed at the pulse overlap time-of-flight (TOF) diffractometer POLDI at the Swiss Spallation Neutron Source (SINQ) at the Paul Scherrer Institut (PSI), Villigen, Switzerland. A greatly simplified sketch of the POLDI measurement principle is shown in Fig. 2 ▸(*c*). POLDI uses a pulsed neutron beam with a 1D ^3^He chamber detector. The detector is TOF and angle sensitive with an angular coverage of 2θ = 75–105°. The signal is integrated over the whole angular range. This implies that the strain component is averaged around ±7.5° from the scattering vector. All measurements were made using the 1.5 × 1.5 mm^2^ full width at half-maximum collimator to define the diffracted beam. The incident beam shape was defined by the slit optics. A *d*_0_-grid was extracted from a sister specimen by electrical discharge machining as depicted in Fig. 2 ▸(*e*). The single cubes have the dimensions 3 × 3 × 3 mm^3^ and are connected in the grid to simplify their alignment. To fully immerse the gauge volume in the cuboids of the *d*_0_-grid, a 2.6 × 2.6 × 1.5 mm^3^ gauge volume was defined for the measurements of *d*_0_ along the three orthogonal directions BD, L and T. However, to obtain sufficient sampling statistics, a 1.5 × 1.5 × 20 mm^3^ matchstick-shaped gauge volume was used to measure *d^hkl^* along BD and T in the prism. For optimization, depending on the path length, the acquisition time was adjusted between 30 and 45 min.

Data analysis was performed using a Gaussian peak function within *Mantid* (Arnold *et al.*, 2014 ▸). Additional information on the experimental setup and the data evaluation of POLDI can be found in the literature (Stuhr, 2005 ▸; Stuhr *et al.*, 2006 ▸, 2005 ▸).

#### 2.3.5. Monochromatic neutron diffraction stress analysis

Bulk residual stress determination was conducted using the KOWARI strain scanner located at the Australian Nuclear Science and Technology Organisation (ANSTO) in Lucas Heights. The principle of the technique is depicted in Fig. 2 ▸(*d*). In contrast to the pulsed white beam at POLDI, a specific wavelength (in our case 1.53 Å) is selected by a silicon monochromator. Using a diffraction angle of 2θ ≃ 90°, a 1.5 × 1.5 × 1.5 mm^3^ gauge volume was defined by slits in the incoming and diffracted beams. The positional accuracy was better than 0.1 mm. The detailed measurement conditions are listed in Table S1. Measurements of *d*_0_^311^ were performed along the L direction of the central cube in the *d*_0_-grids of both H_0°_ and H_45°_. To assess the RS distribution, an equally distributed 8 × 8 point grid was defined in the BD–T cross section of H_0°_ and H_45°_ at the specimen mid-length L = 55 mm, Fig. 2 ▸(*e*)]. In addition to measurements of *d*_0_^311^, the stress balance conditions based on these measured *d* ^311^ values were applied to the T and BD components, using an in-house developed Python script.

The obtained diffraction peaks were fitted using a Gaussian profile and the texture-based analysis of the RS was performed directly in the software *ISODEC* (Gnäupel-Herold, 2012 ▸). The set of linear equations is not overdetermined since we only measured the three orthogonal strain components ɛ_BD_, ɛ_L_ and ɛ_T_. Thus, the error on the stress is estimated by propagating the errors in *d* ^311^ and *d*_0_^311^. Since neutron diffraction knowledge of *d*_0_^311^ is required, the linear equation system must be expressed in the 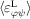 form [see equation (2)].

## 3. Results

### 3.1. Microstructure and texture

**Figure 3:**
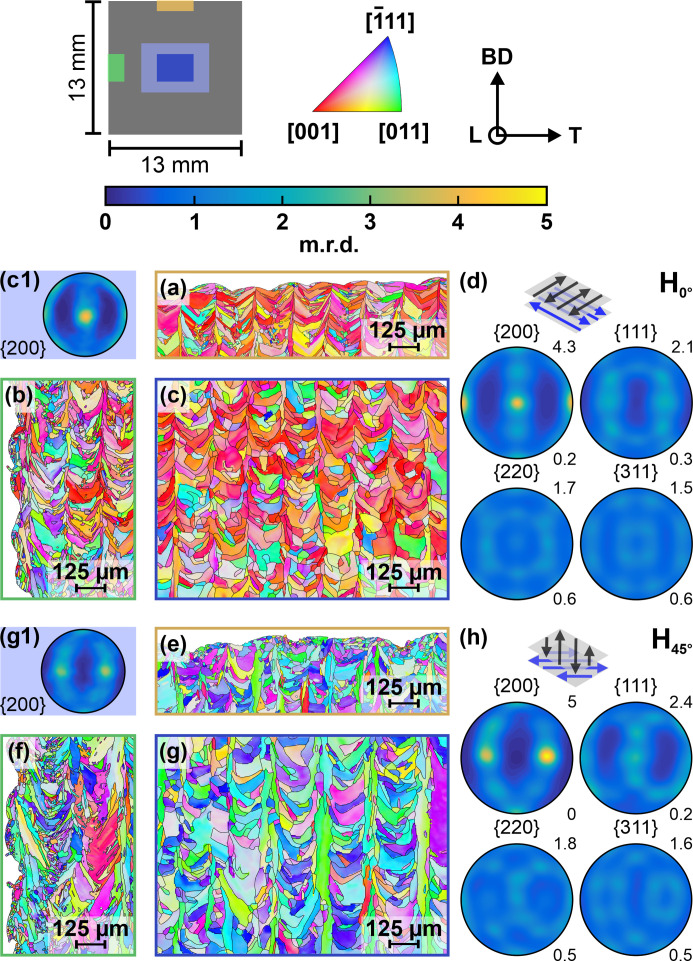
Orientation maps of the samples (*a*)–(*c*) H_0°_ and (*e*)–(*g*) H_45°_ acquired near the top surface [(*a*) and (*e*)], the side surface [(*b*) and (*f*)] and at the center of the cross section (probed area 1.2 × 0.9 mm^2^) [(*c*) and (*g*)]. The view is along the L direction. The {200}-pole figures (probed area 4 × 3 mm^2^) corresponding to (*c*) and (*g*) are shown in (*c*1) and (*g*1). The {200}, {111}, {220} and {311} pole figures acquired via neutron diffraction are shown in (*d*) and (*h*) for H_0°_ and H_45°_, respectively.

The orientation maps viewed along L acquired by EBSD of the specimens H_0°_ and H_45°_ are shown in Figs. 3 ▸(*a*)–3 ▸(*c*) and Figs. 3 ▸(*e*)–3 ▸(*g*). In addition, the calculated {200} pole figures (for the maps acquired on the cross section) are shown in Figs. 3 ▸(*c*1) and 3 ▸(*g*1). The near-surface maps qualitatively reveal that no texture gradient towards the surface exists. However, they also show that the lateral and top surfaces of both H_0°_ [Figs. 3 ▸(*a*) and 3 ▸(*b*)] and H_45°_ [Figs. 3 ▸(*e*) and 3 ▸(*f*)] exhibit a degree of surface roughness, as no contouring was performed during manufacturing. The highest peak-to-valley measure of the surface roughness based on the localized region (*i.e.* statistically very limited) of the EBSD maps in Fig. 3 ▸ is of the order of 70 µm. The neutron [Figs. 3 ▸(*d*) and 3 ▸(*h*)] and EBSD texture measurements [Figs. 3 ▸(*c*1) and 3 ▸(*g*1)] of the bulk yield similar {200} pole figures. In essence, a cube-type texture can be observed in both H_0°_ [Fig. 3 ▸(*d*)] and H_45°_ [Fig. 3 ▸(*h*)] specimens. Since the scanning vectors are aligned with the geometry in H_0°_, the 〈100〉 directions are aligned with the L, T and BD directions. The texture strength of H_0°_ is characterized by the texture index *J*_ODF_(H_0°_) ≃ 1.8. While the texture intensities in the {200} pole figure are equal along L and T, the {220} pole figure shows that a mixed 〈100〉/〈110〉-type texture is present along BD. Even though the 〈100〉/〈110〉-type texture is preserved along BD in H_45°_, the change of the scan pattern causes a 45° rotation of the cube-type texture around BD (*i.e.* 〈110〉/〈111〉-type texture along L and T). This texture is characterized by a texture index *J*_ODF_(H_45°_) ≃ 2.1. EBSD as a surface-specific technique provides spatial resolution to characterize grain morphology and texture. However, the calculation of a representative ODF is limited by the sampling statistics. In this context, the neutron diffraction texture measurements probed the entire volume of the cylinders (∼402 mm^3^), rather than the 4 × 3 mm^2^ area probed by EBSD (the penetration depth of the electron beam is only a few nanometres). Thus, although the textures determined by EBSD and neutron diffraction are in agreement, all subsequent texture-based RS determinations (*i.e.* bulk and surface) use calculated ODFs from neutron texture measurements, because the probed volume is millions of times larger. Such data show the strongest texture and thereby represent the worst case scenario of the influence of crystal orientation on the RS determination.

### 3.2. Stress factors

**Figure 4:**
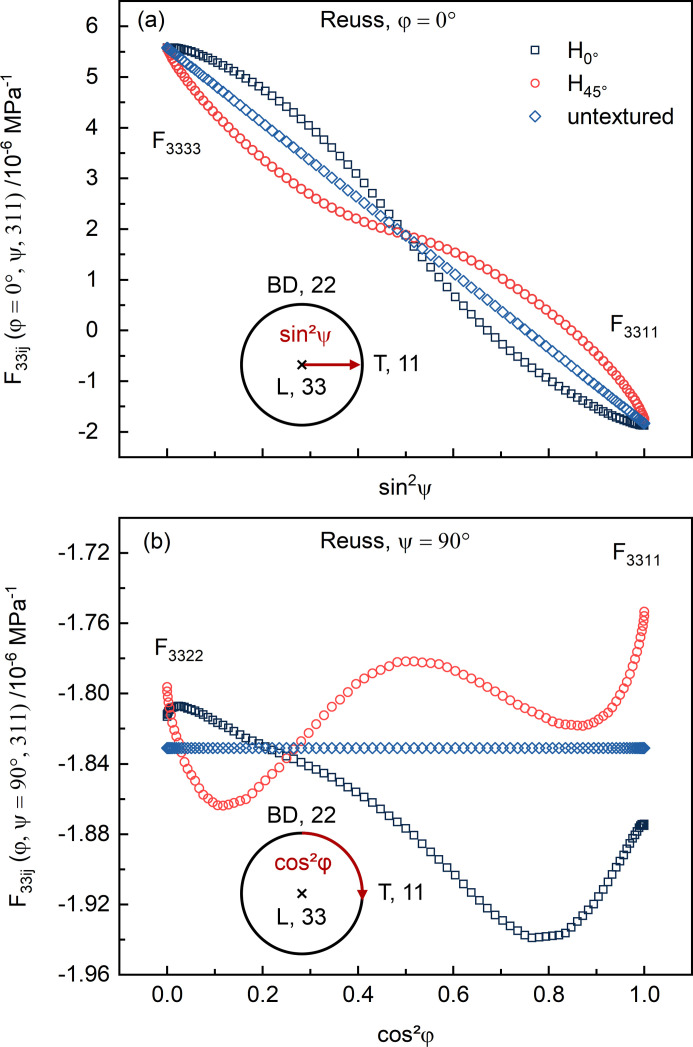
Exemplary comparison of the calculated stress factors (*F*_33*ij*_) showcased for a uniaxial stress acting along the L direction for H_0°_, H_45°_ and a hypothetical untextured sample: (*a*) response in the L–T (*i.e.* 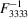 akin to *E^hkl^*) and (*b*) response in the BD–T plane (perpendicular to load axis).

Taking into account the calculated {311} pole figures shown in Figs. 3 ▸(*d*) and 3 ▸(*h*), the stress factors *F*_33*ij*_(φ, ψ, 311) of H_0°_ and H_45°_ are shown in Figs. 4 ▸(*a*) and 4 ▸(*b*) as a function of ψ and φ, respectively. As previously mentioned, the calculations based on the hypothesis of isotropic elasticity are linear combinations of the DECs *s*_1_ and 1/2*s*_2_ and show a linear dependence of *F*_33*ij*_ on sin^2^ψ in the plane containing the load axis. The calculated *F*_33*ij*_ according to the texture-based Reuss model are very different for the two specimens H_0°_ and H_45°_. As an effect of the difference in texture (Fig. 3 ▸), *F*_33*ij*_ is larger for H_0°_ up to sin^2^ψ ≃ 0.5 but smaller above sin^2^ψ ≃ 0.5 [Fig. 4 ▸(*a*)]. Note that the point symmetry of the stress factors in Fig. 4 ▸(*a*) arises from the cube-type texture (see Fig. 3 ▸): the intensity in the {311} pole figures at φ = 0° and ψ = 45° is identical for H_0°_ [Fig. 3 ▸(*d*)] and H_45°_ [Fig. 3 ▸(*h*)]. In principle, the textures of H_0°_ and H_45°_ are akin, just rotated by 45° around the build axis. Therefore, the stress factors are also offset by 45° as they are weighted according to their orientation distribution function. In the plane perpendicular to the applied load, *F*_33*ij*_ is independent of φ for an isotropic material (= *s*_1_), while becoming dependent on φ in the presence of crystallographic texture [Fig. 4 ▸(*b*)].

### 3.3. X-ray diffraction: surface and sub-surface RS

#### 3.3.1. RS before removal from the baseplate

**Figure 5:**
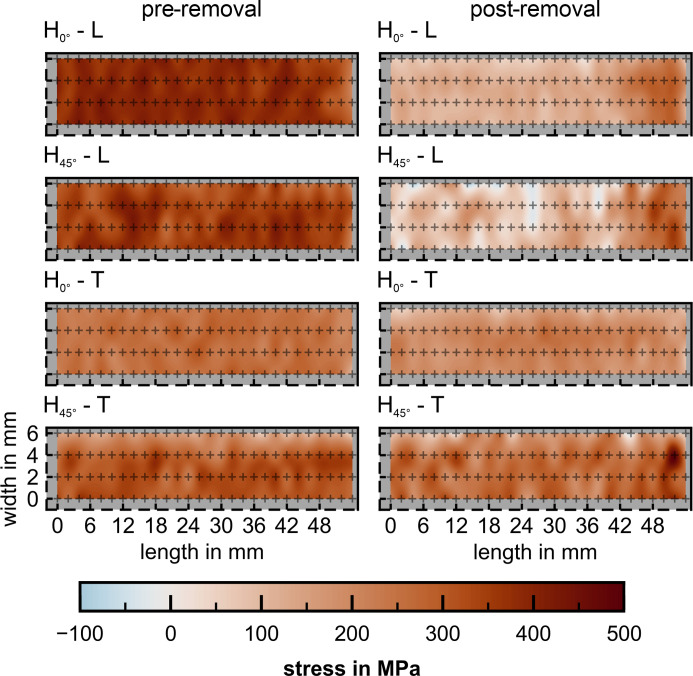
Linearly interpolated contour plots of the laboratory XRD RS measurements on the top surface of the specimens H_0°_ and H_45°_ along their L and T directions. The measurements were performed pre- (average measurement error H_0°_ ≃ 27 MPa, H_45°_ ≃ 34 MPa) and post-removal from the build plate (average measurement error H_0°_ ≃ 29 MPa, H_45°_ ≃ 32 MPa). Measurement positions are highlighted by the crosses and were distributed as depicted in Fig. 2 ▸(*e*). 0,0 is the center of the specimen top surface.

The surface RS maps (L and T directions) for a quarter of the sample surface of the prisms H_0°_ and H_45°_ are depicted in Fig. 5 ▸. The drop in RS close to the specimen edges (width = 6 mm, length = 54 mm) is associated with misalignment. If we ignore these points near the edges, an average maximum stress of 383 ± 28 MPa is present in H_0°_ along L prior to removal from the baseplate. In contrast, a minimum average stress of 255 ± 25 MPa is present in the T direction. For H_45°_ the surface stress appears broadly isotropic, as the average stresses have similar magnitude when considering the error: 355 ± 33 MPa along L and 305 ± 34 MPa along T.

#### 3.3.2. RS after removal from the baseplate

Once the specimens are removed from the baseplate, stress redistribution and relaxation occur, due to distortion in the L direction: the surface longitudinal stress relaxes (from the edge up to L = 42 mm) to an average magnitude of 121 ± 17 MPa (≃ 68% relaxation) and 88 ± 54 MPa (≃ 75% relaxation) for H_0°_ and H_45°_, respectively. However, close to the edges, a higher-magnitude tensile RS of about 240 MPa is present, which introduces a comparable bending moment in the two specimens. Along the T directions, stress redistribution is negligible and only small relaxations of about 55 MPa (≃ 21%) in H_0°_ and 40 MPa (≃ 13%) in H_45°_ are observable.

#### 3.3.3. Determination of sub-surface principal stress

**Figure 6:**
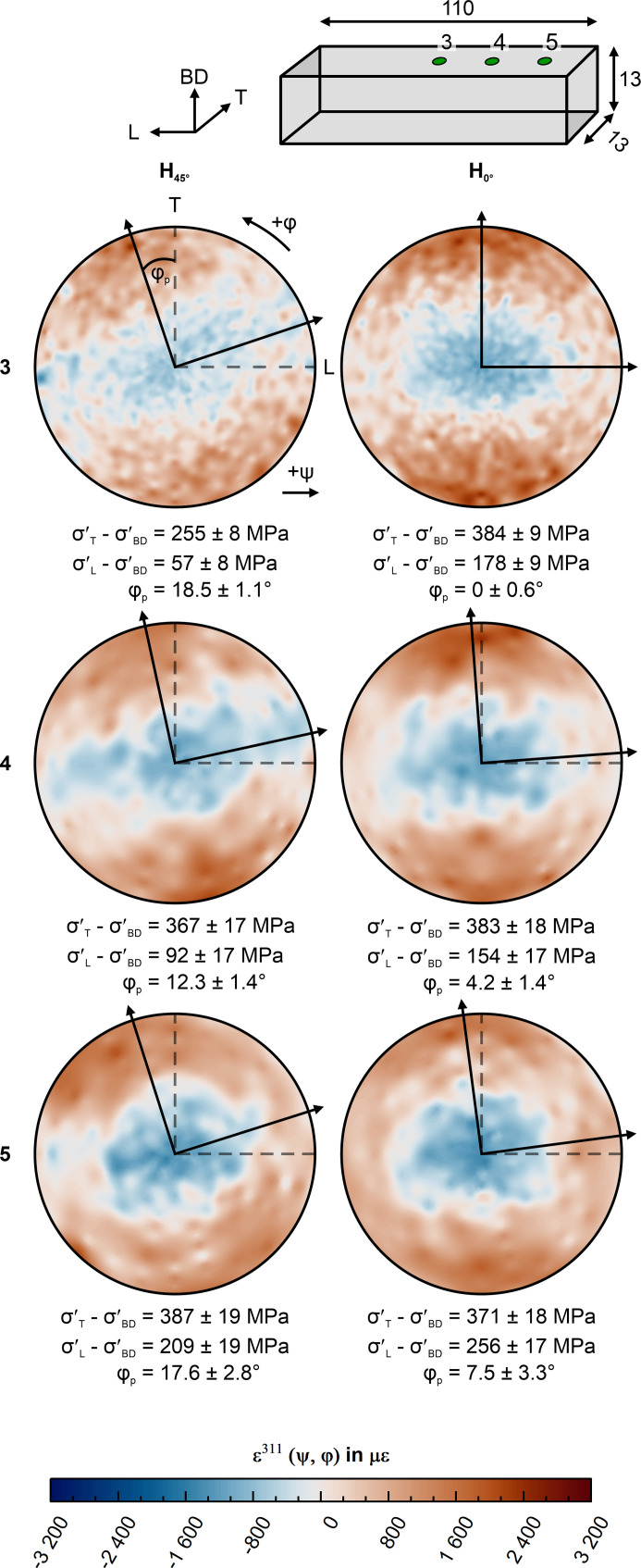
Top surface strain pole figures of measurement points 3–5 calculated for the 311 reflection, where *d*_0_^311^ is defined as the average value of all *d* ^311^ (ψ, φ). The arrows mark the in-plane principal directions according to the eigenvalue decomposition.

**Figure 7:**
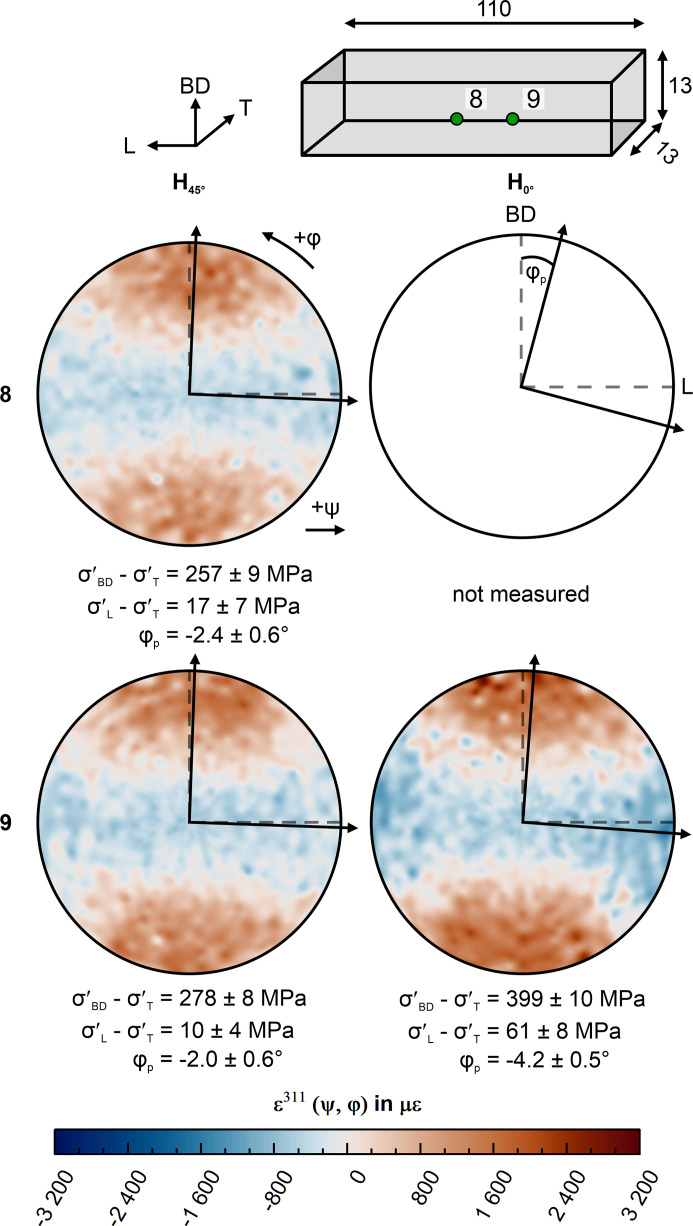
Side surface strain pole figures of measurement points 8 and 9 calculated for the 311 reflection defining the average value of all *d* ^311^(ψ, φ). The arrows mark the in-plane principal directions according to the eigenvalue decomposition.

The strain pole figures acquired at the synchrotron beamline P61A are shown in Figs. 6 ▸ and 7 ▸ for the top (points 3–5) and side surfaces (points 8 and 9), respectively. From these sub-surface strain pole figures, the in-plane principal strain can be directly determined in a qualitative fashion. In all strain pole figures acquired close to the center (*i.e.* points 3, 4, 8, 9), a strain plateau at ±30° in φ is observable around the direction of maximum and minimum strain. This plateau begins to transform into a uniform ‘ring’ of large strain at about 10 mm from the edges (*i.e.* stress state becomes transversely isotropic) of the top surface point 5. This observation is in line with the post-removal XRD measurements (Fig. 5 ▸). Further, the strain pole figures show that the direction of largest sub-surface strain in H_0°_ coincides with the transverse direction T for measurements in the L–T plane (Fig. 6 ▸); the smallest strain (*i.e.* average slope of the ɛ versus sin^2^ψ curve) is found along the longitudinal direction L. In contrast, the strain pole figures of H_45°_ in the L–T plane reveal a rotation of the in-plane sub-surface principal axes around BD towards the geometrical axes (Fig. 6 ▸). Such qualitative observations are confirmed quantitatively by the eigenvalue decomposition results as shown in Fig. 6 ▸. The smaller magnitude of the sub-surface principal stress at measurement point 3 in H_45°_ corresponds to local stress relaxation induced by the layer removal performed on the side surface. In the case of the side surface measurements (7–9), the strain pole figures (Fig. 7 ▸) reveal the alignment of the maximum sub-surface principal strain with BD irrespective of the scanning strategy used. The eigenvalue decomposition reveals an ∼120 MPa larger sub-surface deviatoric principal stress difference σ′_BD_ − σ′_T_ in the H_0°_ specimen than in H_45°_. Also in this case, the stress state is transversely isotropic with respect to BD (*i.e.* the stress difference σ′_L_ − σ′_T_ ≃ 0). The resulting sub-surface principal stress values of all measured points 1–9 can be found in Table S1.

#### 3.3.4. Layer removal

**Figure 8:**
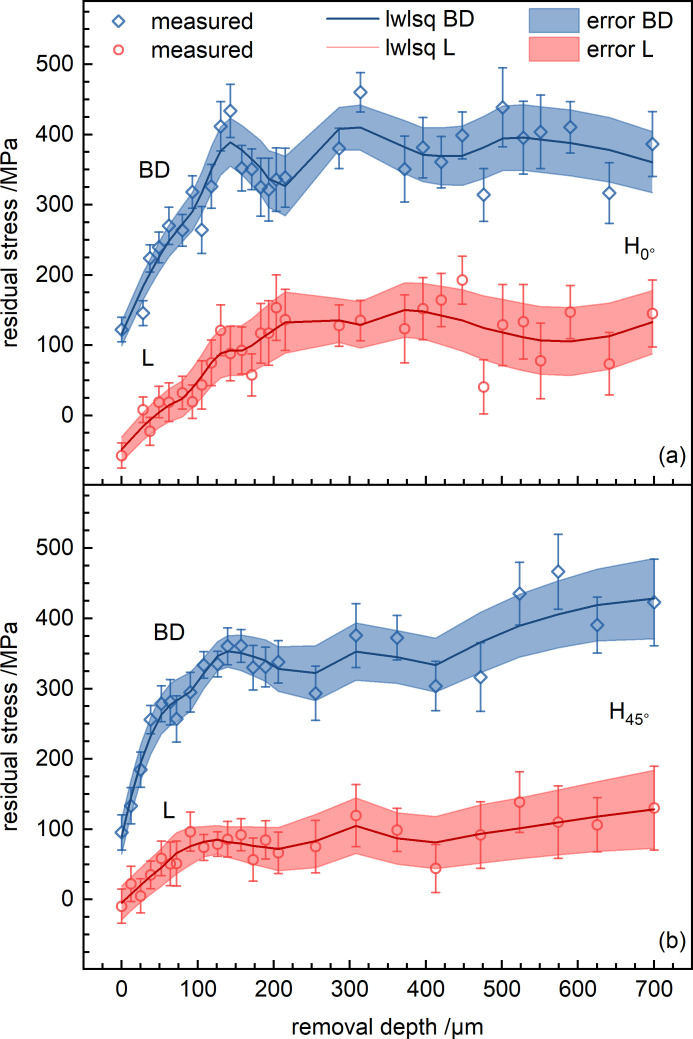
Through-thickness (T) RS profiles obtained by incremental electrolytic layer removal at the center of the side surface (L ≃ 55 mm, BD ≃ 6.5 mm) [see Fig. 2 ▸(*e*)] for the specimens (*a*) H_0°_ and (*b*) H_45°_. No stress relaxation corrections were applied. To guide the reader’s eye, data smoothing has been performed in *OriginLab* by the locally weighted least-squares (lwlsq) method.

Although correction formulae for the determination of RS upon layer removal are available (Moore & Evans, 1958 ▸), for relatively shallow removal depths it is known that the differences of residual stress between the measured and corrected values are negligible. Therefore, Fig. 8 ▸ shows the uncorrected results of the layer-removal method measurements up to a depth of 700 µm (∼5% of the total thickness). For both specimens H_0°_ [Fig. 8 ▸(*a*)] and H_45°_ [Fig. 8 ▸(*b*)], the RS state at the surface is characterized by tensile stresses of small magnitude along BD and around 0 MPa along L. At shallow depths (first 100 µm), an increase of the stress is observed, until a stress plateau of σ_BD_ = 350 MPa and σ_L_ = 100 MPa is reached. This behavior is believed to be connected to the inherent surface roughness of the specimens, as the penetration depth of Mn *K*α radiation in IN718 is small. Even though the scanning strategy was different, the average stress at the plateau appears to be similar in the two specimens. Yet at shallower depths (*e.g.* 125 µm) the maximum stress is larger in H_0°_ (≃ 410 MPa) than in H_45°_ (≃ 330 MPa).

### 3.4. Neutron diffraction: bulk RS

#### 3.4.1. The stress-free reference *d*_0_^311^

**Figure 9:**
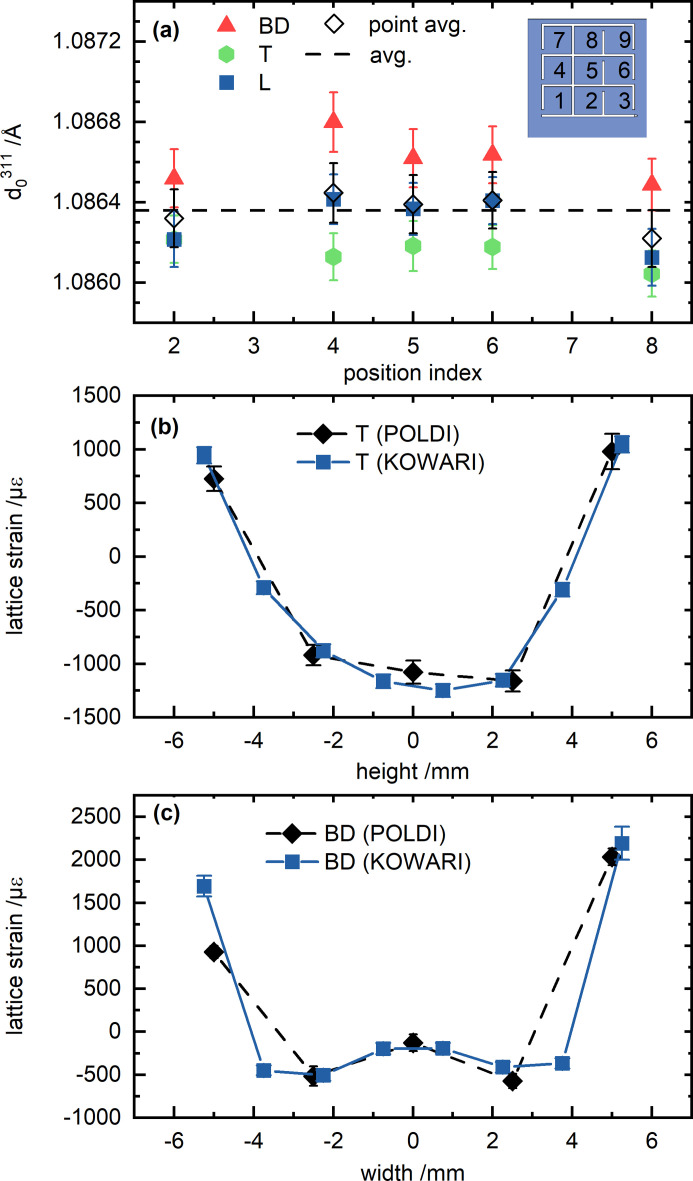
(*a*) *d*_0_^311^ measurements performed at the POLDI beamline in the *d_0_*-grid of specimen H_0°_ according to the coordinate system in Fig. 2 ▸(*e*). Calculated lattice strains for (*b*) T and (*c*) BD, measured along the height and the width in the H_0°_ prism. The strain calculation for the POLDI data is based on a position independent average of *d*_0_^311^ [see dashed line in (*a*)], while the strain calculation for the KOWARI data is based on the value obtained from measuring along L at positional index 5.

**Figure 10:**
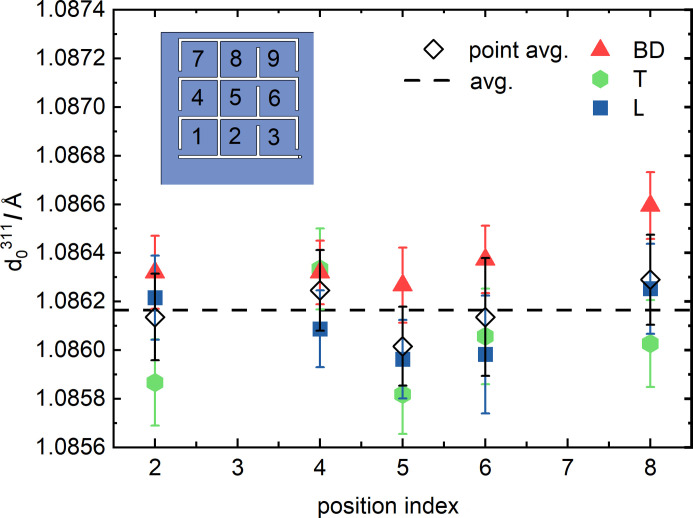
*d*_0_^311^ measurements performed in the d_0_-grid of specimen H_45°_ according to the coordinate system in Fig. 2 ▸(*e*) at the POLDI beamline.

Spatially resolved measurements of *d*_0_^311^ were performed on the *d*_0_-grid (see above) at the POLDI beamline. The results are shown in Fig. 9 ▸(*a*). No clear variation with respect to the build height or transverse direction can be observed for H_0°_ [Fig. 9 ▸(*a*)]. Even though the 2.6 × 2.6 × 1.5 mm^3^ gauge volume used for the POLDI measurements is close to full immersion, different *d*_0_^311^ values were measured in the three directions [Fig. 9 ▸(*a*)]. However, a pointwise average for the three directions corresponds well to the L direction of the measured 3 × 3 × 3 mm^3^ cuboids. The overall average [dashed line in Fig. 9 ▸(*a*)] corresponds to the L direction at positional index 5. This average was used for the calculation of lattice strain from POLDI data for H_0°_. In fact, such strain agrees with the strain determined by KOWARI using the *d*_0_^311^ measured along L at positional index 5 [Figs. 9 ▸(*b*) and 9 ▸(*c*)]. As opposed to H_0°_, the directional spread of *d*_0_^311^ is much smaller in H_45°_, yet the overall average has a slightly worse correlation (although still within the error bar) to the L direction in the center of the *d*_0_-grid (*i.e.* at positional index 5, see Fig. 10 ▸). For the determination of all subsequent bulk RS values (for H_0°_ and H_45°_) from measurements at KOWARI, *d*_0_^311^ along L at positional index 5 is used.

#### 3.4.2. Stress mapping

**Figure 11:**
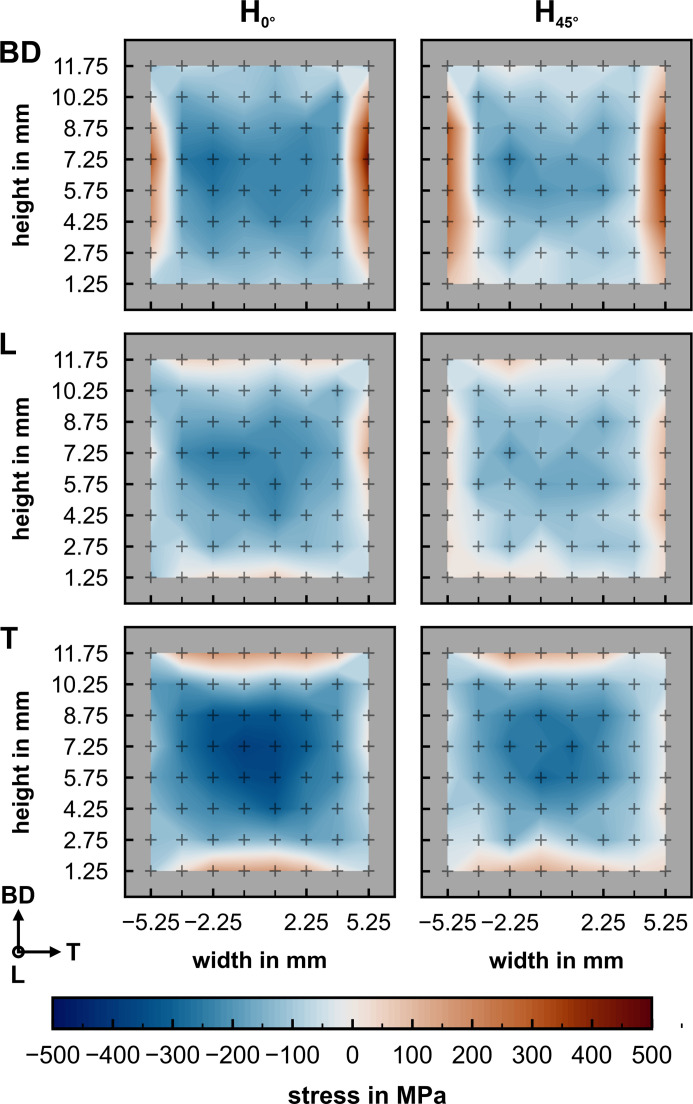
Comparison of the bulk RS maps acquired in the cross section at mid-length on the KOWARI strain scanner at ANSTO using the measured *d*_0_^311^ along L of the respective specimens H_0°_ and H_45°_. The average measurement error is 27 MPa for H_0°_ and 32 MPa for H_45°_.

The RS maps acquired at the strain scanner KOWARI in the cross sections displayed in Fig. 2 ▸(*e*) are shown in Fig. 11 ▸. It is evident from these measurements that the tensile RS close to the surface is balanced by compressive stress in the bulk. Furthermore, a slight asymmetry in the stress maps from left to right can be observed. The stress relaxation on removal from the baseplate results in a low stress (about 50 MPa) along L close to the sample surface (center of gauge volume 1.25 mm below the surface) in both specimens. Overall, the RS distributions look alike, except for a larger compressive stress preserved in the H_0°_ specimen.

## 4. Discussion

### 4.1. Influence of preferred grain orientation

**Figure 12:**
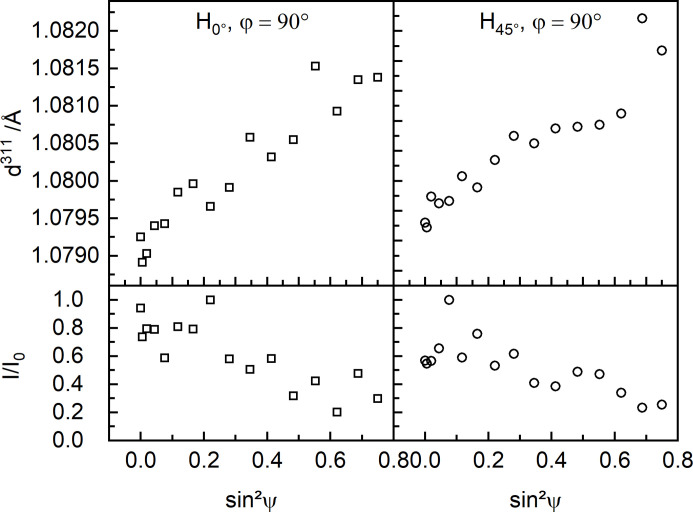
*d* ^311^–sin^2^ψ distributions and respective relative intensities of H_0°_ and H_45°_ measured in the BD–L plane at φ = 90° (ψ tilting towards BD) at measurement position 9 [see Fig. 2 ▸(*e*)], showing evidence of crystallographic texture.

In theory, the presence of crystallographic textures invalidates the use of methods based on the hypothesis of isotropic elastic behavior. Yet, in most cases the hypothesis of isotropic elastic constants is used. This holds true even though it is known that crystallographic texture is present in PBF-LB/M/IN718 manufactured specimens (Volpato *et al.*, 2022 ▸). Fig. 12 ▸ shows the *d* ^311^–sin^2^ψ curves and their relative intensities at φ = 90° in the BD–L plane for H_0°_ and H_45°_. In a case without preferred orientation, such a distribution should exhibit linearity (Vanhoutte & Debuyser, 1993 ▸). In addition, the relative intensity should be nearly independent of ψ (Spieß *et al.*, 2009 ▸), yet gradually decrease at higher ψ angles due to the grazing incidence. Instead, both *d* ^311^–sin^2^ψ curves are nonlinear (especially for H_45°_), *i.e.* show clear evidence of crystallographic texture. Such a nonlinearity has been recently observed for the {311} lattice planes in PBF-LB/M/IN718 (Mishurova *et al.*, 2018 ▸; Serrano-Munoz, Fritsch *et al.*, 2021 ▸), although the {311} lattice planes are supposed to behave in an isotropic manner. In fact, Mishurova *et al.* (2018 ▸) and Serrano-Munoz, Fritsch *et al.* (2021 ▸) determined the RS using a linear fit. Although Mishurova *et al.* (2018 ▸) and Serrano-Munoz, Fritsch *et al.* (2021 ▸) proved this to be a fair approximation, this approach may lead to significant errors in the calculation of RS, when compared with approaches fitting nonlinear functions (*i.e.* those considering texture) to *d*–sin^2^ψ curves (Vanhoutte & Debuyser, 1993 ▸).

**Figure 13:**
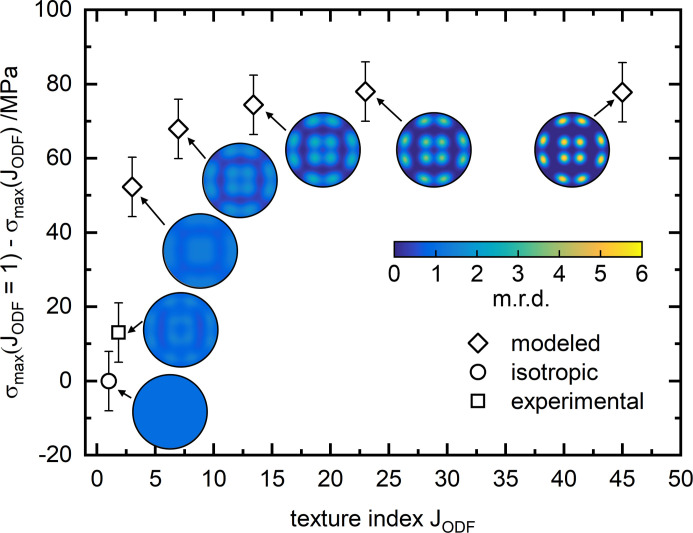
Influence of experimental and modeled {311} pole figures on the calculated residual stress difference compared with the isotropic case of H_0°_ as determined by energy dispersive diffraction at measurement position 3 (see Figs. 2 ▸ and 6 ▸).

To quantify the difference between texture-based and isotropic calculations, we used both isotropic and texture-based calculations within *ISODEC*; the results are outlined in Table 1 ▸. The differences in the obtained RS are small and well within the error bar of the measurements. This corroborates the assumption made by Mishurova *et al.* (2018 ▸), Serrano-Munoz, Fritsch *et al.* (2021 ▸) and Thiede *et al.* (2018 ▸). Most probably, the mild texture of the 311 reflection (maximum 1.6 m.r.d.) has a rather minor effect on the calculated RS and one could still use the hypothesis of isotropic elastic constants. In fact, the isotropic and texture-based calculations of the neutron diffraction and energy-dispersive RS data are comparable for H_0°_. However, when stronger cube-type textures are modeled in MTEX and accounted for in the texture-based analysis of H_0°_, the absolute stress difference between isotropic and texture-based calculations increases up to 80 MPa (Fig. 13 ▸). This difference is well beyond the error bar of the determination and is above 15% of the actual stress value. Especially since in high-power PBF-LB (1000 W) strong cube-type textures (*t* ≃ 20) are realized (Zhong *et al.*, 2023 ▸), texture-based methods should be employed in such a case. However, for texture indices *J*_ODF_ < 3, the effect of texture on the RS values seems to rapidly decrease (Fig. 13 ▸). It must be emphasized that this observation is based on modeled textures applied to experimental data possessing much lower crystallographic texture. In reality, it is practically impossible to produce material with different textures yet the exact same residual stress field using PBF-LB.

Therefore, the general assumptions used for a diffraction-based analysis of RS must be checked on a case-by-case basis (low texture factor, columnar grain shape). Whenever the ODF is known, the use of texture-based methods for the determination of RS is recommended. Once different reflections are used for RS analysis (*e.g.* energy-dispersive methods), texture-based approaches become unavoidable.

### 4.2. The scanning strategy determines the RS distribution

Several studies reporting surface RS distributions have shown that longer scan vectors lead to higher tensile RS in Ti6Al4V (Kruth *et al.*, 2012 ▸; Ali *et al.*, 2018 ▸) and IN718 (Serrano-Munoz, Ulbricht *et al.*, 2021 ▸). Furthermore, it is known that the larger principal residual stress is always parallel to the track of the scan direction in the final deposited layer while the specimen is attached to the baseplate for PBF-LB/M/Ti64 (Levkulich *et al.*, 2019 ▸). In contrast, Bayerlein *et al.* (2018 ▸) showed (for an unspecified scanning strategy) that the principal directions are approximately aligned in the direction of the sample edges for as-built PBF-LB/M/IN718 cuboids. Similar observations have been made for PBF-LB/M/IN625 structures, where the principal direction coincides with the main geometrical axis of the structure (Fritsch *et al.*, 2021 ▸). However, Fritsch *et al.* (2021 ▸) showed that the determination of the principal stress is only independent of the choice of the measurement directions if one uses nine directions.

These observations seem to be transferrable to PBF-LB/M/IN718. In H_0°_ the scanning vector was oriented along the length (110 mm) and width (13 mm) of the rectangular prism for alternate layers. The largest stress along the L direction in H_0°_ (Fig. 5 ▸) can thus be explained by the larger thermal gradient when scanning along this direction: in fact, the aspect ratio between the scan length of alternate layers is about 7. If the scan vectors become of equal length, as in the case of a 45° rotation to the geometrical axis in H_45°_, the surface RS magnitudes along T and L become similar. Further, the scanning strategy influences the orientation of the surface principal stress axes relative to the geometrical axis. It is hypothesized that, prior to removal from the baseplate, the scanning direction dictates the sub-surface principal stress direction (∼45° to L and T in H_45°_). This would explain the equivalent surface RS values along the L and T directions prior to removal: both L and T lie at 45° from the principal axis.

### 4.3. RS redistribution on removal from the baseplate

In agreement with the present work, Thiede *et al.* (2018 ▸) found a similar relaxation pattern of the surface RS in horizontally manufactured IN718 prisms (with a rounded tip). Prior to removal from the baseplate, the surface RS had high tensile magnitude with insignificant changes across the specimen surface. On removal from the baseplate, an overall relaxation with a steep increase of the surface RS in the longitudinal direction towards the tip was found, irrespective of the scanning strategy applied [see also Serrano-Munoz, Ulbricht *et al.* (2021 ▸)]. In contrast to our work, Thiede *et al.* (2018 ▸) observed the surface RS in the transverse direction to be the largest prior to removal and it additionally showed significant relaxation. However, both the specimen cross section (20 × 20 mm^2^) and the stripe-wise scanning strategy were substantially different compared with this study. Therefore, the disagreement with the present study outlines the influence of such aspects on the surface RS distribution.

Additionally, the surface RS values reported by Thiede *et al.* (2018 ▸) were significantly higher than those observed in our study. On the one hand this is connected to the choice of a Kröner-type grain-interaction model [see also Pant *et al.* (2020 ▸)]. In fact, Serrano-Munoz, Ulbricht *et al.* (2021 ▸) showed that the use of the Reuss model for similar specimens yields a more sensible magnitude of surface and sub-surface RS. On the other hand, this is – to a degree – also dependent on the geometry and the process parameters [*i.e.* the scanning strategy (Nadammal *et al.*, 2021 ▸)]. Distortion measurements of this kind of sample geometry show that the sample tends to deform towards the tip (Thiede *et al.*, 2018 ▸; Mishurova *et al.*, 2018 ▸; Serrano-Munoz, Fritsch *et al.*, 2021 ▸). In addition, the distortion tends to be somewhat dependent on the scanning strategy (Serrano-Munoz, Ulbricht *et al.*, 2021 ▸).

Our synchrotron experiments reveal that the sub-surface principal axes are aligned with the geometry if the scanning vectors are alternatingly parallel to L and T. However, the principal directions in the L–T plane are rotated by ∼13° from the main geometrical axes if the scanning vectors are oriented 45° to the geometry. This indicates that a ‘back rotation’ of the sub-surface principal components around BD occurs, due to the distortion on removal from the baseplate. This last finding would explain the similarity of the surface RS for H_0°_ and H_45°_ after removal from the baseplate (Fig. 5 ▸). The inherent distortion causes the geometry to influence the sub-surface principal direction. As a consequence, the slight rotation, in conjunction with the strain plateau of ±30°, results in similar RS values along the geometrical axes. This is emphasized by the negligible difference between the sub-surface deviatoric stress along T (σ_T_ − σ_BD_ = 373 ± 13 MPa) and the maximum principal components for H_45°_ (σ′_T_ − σ′_BD_ = 381 ± 18 MPa) measured at point 2. In neutron diffraction measurements, the detectors typically average over a range of ±15° (±7.5° from the diffraction vector). This average implies that any small difference between geometrical and stress axes would not influence the RS values.

On the other hand, one of the sub-surface principal stress axes always remains aligned with BD irrespective of the scanning strategy. In fact, the laser beam parameters predominantly determine the RS distribution along BD, rather than the scanning strategy. This results in similar distortion along BD for different scanning strategies.

### 4.4. On the choice of the stress-free reference

A critical point of uncertainty for the determination of the bulk RS by neutron diffraction techniques may arise from inaccuracy of the stress- (or strain)-free reference (Withers *et al.*, 2007 ▸). It has thus been proposed to utilize different methods to cross-check *d*_0_^*hkl*^ values (Withers *et al.*, 2007 ▸). In fact, the cross-check between mechanically relaxed cubes and the application of theoretical boundary conditions such as the stress balance yields a suitable sanity check for the measured *d*_0_^*hkl*^ values. However, for the applicability of the stress balance method it must be ensured that no spatial variation of *d*_0_^*hkl*^ exists within the cross section of interest (Withers *et al.*, 2007 ▸). In the case of PBF-LB it has been shown that no large variations of *d*_0_^*hkl*^ across the specimen occur (Serrano-Munoz *et al.*, 2022 ▸; Bayerlein *et al.*, 2018 ▸), at least when significant heat concentrations are avoided (Capek *et al.*, 2022 ▸). This has also been observed for the specimens in this work (Fig. 9 ▸). The *d*_0_^311^ values calculated from the application of the stress balance condition to bulk data are listed in Table 2 ▸. The use of the stress-balance-based *d*_0_^311^ (instead of the one based on measurements of the coupons) would shift the calculated stress by about 70 MPa for H_0°_ and 30 MPa for H_45°_. This may be because the surface RS was not included in the stress balance. In fact, if the surface RS (accounting for the surface roughness) is not included in the stress balance, a deviation between experimentally measured and theoretical *d*_0_^311^ occurs (Serrano-Munoz *et al.*, 2022 ▸). The use of the post-removal RS values of the relaxed surface (Fig. 5 ▸) should shift the stress-balance-based *d*_0_^311^ to smaller values. Although no spatial gradient of the experimentally determined *d*_0_^311^ exists, a directional dependence is evident (Fig. 9 ▸). Such a directional dependence has been reported by other researchers for PBF-LB/M/IN718 (Bayerlein *et al.*, 2018 ▸; Thiede *et al.*, 2018 ▸) and PBF-LB/M/316L (Ulbricht *et al.*, 2020 ▸). This direction dependence might arise from possible retention of macroscopic (if the gauge volume is not fully immersed in the cuboid) or intergranular stress (Withers *et al.*, 2007 ▸). As we lack evidence of the cause of this directional dependence, we considered the global average of all *d*_0_^311^ as an appropriate value. Yet, the fact that the gauge volume was close to full immersion implies the prevalence of intergranular over macro stress. If one accounts for the directional dependence of *d*_0_^311^ [Fig. 9 ▸(*a*)], the RS values would shift in H_0°_ but not in H_45°_ (the directional variation is much smaller, see Fig. 10 ▸). Finally, the similarity between the XRD-based (where no precise *d*_0_^311^ is required) and neutron-diffraction-based RS strongly indicates that the direction-independent *d*_0_^311^ of the L component (being similar to the overall average) is appropriate in this special case.

### 4.5. Through-thickness stress distribution

A critical point of the stress profile within PBF-LB manufactured alloys is the distribution close to the surface. Overall, the increase of the RS magnitudes in the sub-surface region can be linked to the surface roughness of the parts, since the roughness contributes to a stress relaxation in the vicinity of the surface (Serrano-Munoz *et al.*, 2022 ▸). In fact, the mean roughness of PBF-LB specimens manufactured without a contouring parameter set is reported to be in the range 10–25 µm (Fritsch *et al.*, 2022 ▸; Mishurova *et al.*, 2019 ▸; Sprengel *et al.*, 2022 ▸). It is further known that high tensile stresses are usually present in the sub-surface region (Bayerlein *et al.*, 2018 ▸; Serrano-Munoz *et al.*, 2022 ▸; Serrano-Munoz, Fritsch *et al.*, 2021 ▸; Serrano-Munoz, Ulbricht *et al.*, 2021 ▸; Busi *et al.*, 2021 ▸). The layer removal plus the XRD experiments we performed revealed a sub-surface stress plateau rather than a peak stress. Interestingly, such behavior has also been found by Serrano-Munoz *et al.* (2022 ▸) using neutron diffraction. Therefore, additional sample preparation (*e.g.* electro polishing) or use of high-energy XRD techniques is recommended to overcome such surface roughness effects (Mishurova *et al.*, 2019 ▸).

**Figure 14:**
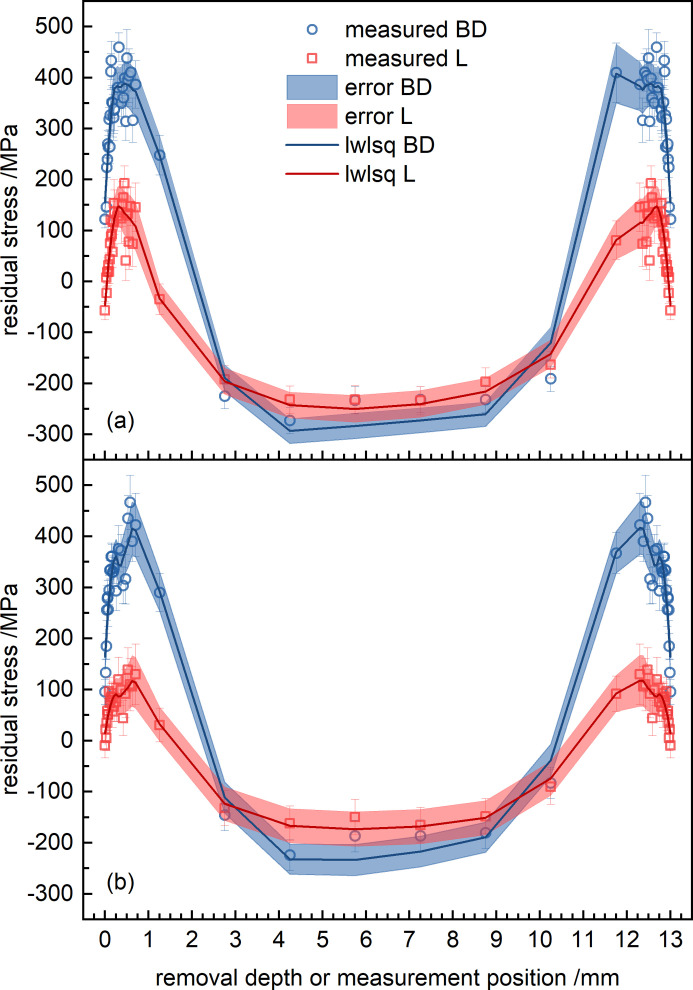
Through-thickness stress profiles of the specimens (*a*) H_0°_ and (*b*) H_45°_; the uncorrected layer removal data are combined with neutron diffraction, assuming symmetry of the layer removal depth profiles.

Fig. 14 ▸ shows the through-thickness stress profiles for the BD and L components of the specimens H_0°_ and H_45°_, combining surface XRD (layer removal) and bulk neutron data. The full profiles are drawn assuming symmetry of the surface and sub-surface RS with respect to the sample center point. It becomes apparent that a strong RS gradient must be present at depths of 0.7–2.75 mm. In this context, Serrano-Munoz *et al.* (2022 ▸) recently showed that the RS decreased at 1.4 mm depth from the lateral surfaces in a 20 × 20 mm^2^ cross section prism produced with a 67°-rotation scan strategy. However, Serrano-Munoz *et al.* (2022 ▸) showed that the plateau below 1.4 mm displayed higher RS compared with our study. First and foremost, the build-up of RS in PBF-LB/M/IN718 is known to depend on the build height [much larger for Serrano-Munoz *et al.* (2022 ▸) than in the present study]: the addition of new layers produces tensile stress in the material directly below (Bayerlein *et al.*, 2018 ▸). In fact, Pant *et al.* (2020 ▸) reported that the magnitude of RS depends on the build orientation of L-shaped specimens produced with a 13° interlayer rotation. The horizontally built specimen (10 mm build height) showed the lowest magnitudes of residual stress, while the largest magnitudes were found for the vertical build orientation (build height 55 mm). Secondly, the use of up-skin (also referred to as contouring) processing is known to cause higher RS magnitudes in Ti6Al4V (Artzt *et al.*, 2020 ▸). While the rotation scanning strategy used by Serrano-Munoz, Ulbricht *et al.* (2021 ▸) would lead to lower RS values compared with other scanning strategies, the effect of the contour and the addition of layers prevails in the present case. Interestingly, the RS profiles observed by Pant *et al.* (2020 ▸) show a similar distribution in their horizontally built specimen: tensile stress is present at the side surfaces along BD, while it is observed along the short direction at the top surface of the structure. Note that the RS values reported by Pant *et al.* (2020 ▸) are not metrologically comparable to our study, because the diffraction elastic constants were calculated using the Kröner model.

## 5. Conclusions

This work discusses the texture-based determination of residual stress in as-built PBF-LB Inconel 718 prisms. Different crystallographic textures were obtained by employing different scanning strategies. Scan vectors aligned with the specimen geometrical axes resulted in 〈100〉 in-plane texture. In contrast, those rotated by 45° to these axes, while maintaining the 90° interlayer rotation, resulted in 〈111〉/〈110〉 in-plane texture. Residual stress determination was performed by utilizing laboratory XRD methods and employing stress factors to account for the specimen texture. Additional laboratory X-ray (layer removal) and neutron diffraction measurements provided further insight into the residual stress distribution after removal from the baseplate. Furthermore, sub-surface principal stress was assessed by energy-dispersive synchrotron diffraction. The consequences of the presence of crystallographic texture on the residual stress determination were studied for both surface- and bulk-related measurements. The following conclusions can be drawn:

(1) Under the conditions used in this study (texture indices < 3), the preferred grain orientation (*i.e.* the crystallographic texture) has a negligible influence on the determined residual stress values. We identified that the high multiplicity of the 311 reflection, its propensity to exhibit mild texture intensities when compared with other reflections (*e.g.* 200) and its quasi-isotropic elastic behavior produce such a result.

(2) Significant redistribution and relaxation of the residual stress (both bulk and surface) occur after the removal from the baseplate. Prior to removal, the longitudinal residual stress is the highest if the scan vectors are aligned with the sample geometrical axes, but longitudinal and transverse stress components become similar when the scan vectors are rotated by 45°. After removal, the residual stress redistributes in such a way that the longitudinal stress relaxes and a bending moment is induced in the specimens. On the other hand, the transverse component barely shows any signs of relaxation or redistribution.

(3) Post-removal synchrotron XRD measurements in the plane perpendicular to the build direction revealed an alignment of the sub-surface stress tensor principal axes with the geometrical axes if the scan vectors are aligned with the geometrical axes. In contrast, the sub-surface stress tensor principal axes rotate around the build direction when the scan vectors are aligned by 45° to the geometry. This rotation seems to be influenced by the residual stress redistribution on removal from the baseplate. Furthermore, the sub-surface strain does not vary as a function of angle around the principal axes; therefore, determining bulk residual stress using measurements along the geometrical axes does not induce large errors.

(4) The combination of laboratory X-ray and neutron diffraction allowed further insight into the residual stress formation and spatial distribution: irrespective of the scanning strategy, similar residual stress distributions after removal from the baseplate were found. By combination of X-ray electrolytic layer removal and neutron diffraction data, the through-thickness stress profile was successfully determined, revealing a sub-surface tensile plateau balanced by compressive stress in the bulk.

## 6. Data availability

Datasets generated and/or analyzed during the current study are available from the corresponding author on reasonable request.

## Supplementary Material

Supporting tables. DOI: 10.1107/S1600576723004855/xx5022sup1.pdf

## Figures and Tables

**Table 1:** Maximum and average values of the RS difference between calculations neglecting (σ_isotropic_) and considering (σ_textured_) crystallographic texture for the measurements at KOWARI Errors represent the standard deviation of all measurements.

Specimen	H_0°_	H_45°_
Maximum (σ_isotropic_ − σ_textured_)	5 MPa	11 MPa
Average (σ_isotropic_ − σ_textured_)	1 ± 1 MPa	2 ± 3 MPa

**Table 2:** Comparison of measured and calculated (boundary condition of BD, T) *d*_0_^311^ values of the L component of the specimens H_0°_ and H_45°_

KOWARI	H_0°_	H_45°_
*d*_0_^311^ measured (Å)	1.08089 ± 3 × 10^−5^	1.08068 ± 4 × 10^−5^
*d*_0_^311^ calculated (Å)	1.08074	1.08061
Δɛ^311^ (μɛ)	−136	−62
Δσ^311^ (MPa)	−71	−32
