# Supplementary material for: Texture-based residual stress analysis of laser powder bed fused Inconel 718 parts
Source: J Appl Crystallogr. 2023 Jun 30;56(Pt 4):1076–90. doi: 10.1107/S1600576723004855 (PMC10405581; doi:10.1107/S1600576723004855)
Supplement: Supplementary file 1 [file j-56-01076-sup1.pdf]

JOURNAL OF
APPLIED
CRYSTALLOGRAPHY

Volume 56 (2023)

Supporting information for article:

Texture-based residual stress analysis of laser powder bed fused Inconel 718 parts

Jakob Schröder, Alexander Evans, Vladimir Luzin, Guilherme Abreu Faria, Sebastian Degener, Efthymios Polatidis, Jan Čapek, Arne Kromm, Gleb Dovzhenko and Giovanni Bruno

***Table S1**: Overview of the measurement conditions for the different diffraction techniques.*

**Technique**	**radiation**	**reflection Diffraction angle**	**ψ/χ-tilting (steps)**	**φ-tilting (steps)**	**assumptions**
Laboratory XRD Xstress G3	Monochromatic, Mnk_{α}	Ni-311 2θ ≈ 156°	-45° to 45° (19)	0°, 90°	Top: $\sigma_{i3} = 0$ $\sigma_{12} = 0$ Side: $\sigma_{i1} = 0$ $\sigma_{23} = 0$
Synchrotron XRD P61A	Energy dispersive, 30 - 200 keV	Ni - 311 E ≈ 55 keV 2θ ≈ 11.946°	0 to 80° (20)	0 - 360° 3, 7, 8 ,9 (5°) 1, 2, 4, 5 (15°)	-
Neutron diffraction POLDI	Time of flight, Q ≈ 1-8 $2\pi d^{-1}$	2θ ≈ 90° ± 15°	2 orthogonal directions (BD, T)		-
Neutron diffraction KOWARI	Monochromatic, λ = 1.53 Å	Ni-311 2θ ≈ 90°	3 orthogonal directions (BD, T, L)		

Table S2: *Obtained eigenvalues for the top surfaces of the specimen $H_{0°}$ and $H_{45°}$ according to the measurement positions 1-9 acquired by energy dispersive synchrotron diffraction.*

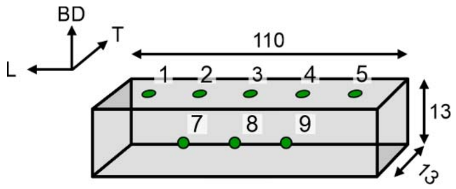

$H_{45°}$	$\#d^{311}$	$\sigma'_T-\sigma'_{BD}$ /MPa	$\sigma'_L-\sigma'_{BD}$ /MPa	φ_p /°	$H_{0°}$	$\#d^{311}$	$\sigma'_T-\sigma'_{BD}$ /MPa	$\sigma'_L-\sigma'_{BD}$ /MPa	φ_p /°
**1**	381	414 ± 18	265 ± 18	21.9 ± 3.6	**1**	384	392 ± 20	300 ± 20	8.3 ± 7
**2**	382	381 ± 18	46 ± 17	8.8 ± 1.1	**2**	384	393 ± 19	172 ± 19	-1.0 ± 0.8
**3**	1863	255 ± 8	57 ± 8	18.5 ± 1.1	**3**	1833	384 ± 9	178 ± 9	0 ± 0.6
**4**	382	367 ± 17	92 ± 17	12.3 ± 1.4	**4**	384	383 ± 18	154 ± 17	4.2 ± 1.4
**5**	377	387 ± 19	209 ± 19	17.6 ± 2.8	**5**	383	371 ± 18	256 ± 17	7.5 ± 3.3
**$H_{45°}$**	**$\#d^{311}$**	**$\sigma'_{BD}-\sigma'_T$ /MPa**	**$\sigma'_L-\sigma'_T$ /MPa**	**φ_p /°**	**$H_{0°}$**	**$\#d^{311}$**	**$\sigma'_{BD}-\sigma'_T$ /MPa**	**$\sigma'_L-\sigma'_T$ /MPa**	**φ_p /°**
**7**	1511	265 ± 9	12 ± 7	-0.5 ± 0.7	**7**	1512	391 ± 10	96 ± 9	-1.3 ± 0.5
**8**	1512	257 ± 9	17 ± 7	-2.4 ± 0.6	**8**	-	-	-	-
**9**	1512	278 ± 8	10 ± 4	-2.0 ± 0.6	**9**	1512	399 ± 10	61 ± 8	-4.2 ± 0.5
